# Trajectories of the Hippocampal Subfields Atrophy in the Alzheimer’s Disease: A Structural Imaging Study

**DOI:** 10.3389/fninf.2019.00013

**Published:** 2019-03-22

**Authors:** Weina Zhao, Xuetong Wang, Changhao Yin, Mengfei He, Shuyu Li, Ying Han

**Affiliations:** ^1^Department of Neurology, Xuanwu Hospital of Capital Medical University, Beijing, China; ^2^Department of Neurology, Mudanjiang Medical University Affiliated Hongqi Hospital, Mudanjiang, China; ^3^School of Biological Science and Medical Engineering, Beihang University, Beijing, China; ^4^Beijing Advanced Innovation Center for Biomedical Engineering, Beihang University, Beijing, China; ^5^Institute of Alzheimer Disease, Beijing Institute for Brain Disorders, Beijing, China; ^6^Institute of Geriatrics, Beijing Hospital, Beijing, China; ^7^National Clinical Research Center for Geriatric Disorders, Beijing, China

**Keywords:** Alzheimer’s disease, amnestic mild cognitive impairment, subjective cognitive decline, magnetic resonance imaging, hippocampal subfields

## Abstract

**Background:**

The hippocampus and hippocampal subfields have been found to be diversely affected in Alzheimer’s Disease (AD) and early stages of Alzheimer’s disease by neuroimaging studies. However, our knowledge is still lacking about the trajectories of the hippocampus and hippocampal subfields atrophy with the progression of Alzheimer’s disease.

**Objective:**

To identify which subfields of the hippocampus differ in the trajectories of Alzheimer’s disease by magnetic resonance imaging (MRI) and to determine whether individual differences on memory could be explained by structural volumes of hippocampal subfields.

**Methods:**

Four groups of participants including 41 AD patients, 43 amnestic mild cognitive impairment (aMCI) patients, 35 subjective cognitive decline (SCD) patients and 42 normal controls (NC) received their structural MRI brain scans. Structural MR images were processed by the FreeSurfer 6.0 image analysis suite to extract the hippocampus and its subfields. Furthermore, we investigated relationships between hippocampal subfield volumes and memory test variables (AVLT-immediate recall, AVLT-delayed recall, AVLT-recognition) and the regression model analyses were controlled for age, gender, education and eTIV.

**Results:**

CA1, subiculum, presubiculum, molecular layer and fimbria showed the trend toward significant volume reduction among four groups with the progression of Alzheimer’s disease. Volume of left subiculum was most strongly and actively correlated with performance across AVLT measures.

**Conclusion:**

The trend changes in the hippocampus subfields and further illustrates that SCD is the preclinical stage of AD earlier than aMCI. Future studies should aim to associate the atrophy of the hippocampal subfields in SCD with possible conversion to aMCI or AD with longitudinal design.

## Introduction

The pathophysiological process of Alzheimer’s disease (AD) is a neurodegenerative disorder characterized by cognitive decline, which is thought to have begun many years before the diagnosis. With the disease progression, as the preclinical AD, subjective cognitive decline (SCD) have worse cognition than normal controls (NC), while objective examination shows that they have not yet reached the level of amnestic mild cognitive impairment (aMCI) or AD dementia (Molinuevo et al., 2017). The main manifestation of SCD is the decline in memory rather than other domains of cognition. It is formally proposed and standardized by Subjective Cognitive Decline Initiative (SCD-I) in a conceptual framework for research on subjective cognitive decline (Jessen et al., 2014). After adjustment for age, sex and education, the stage of neuropsychological examination below threshold was mild cognitive impairment (MCI) or prodromal AD (Petersen et al., 2018). Subsequently, if there are significant interferences in the ability of work or daily activities, cognitive decline progresses onward to the stage of AD dementia (Sperling et al., 2011; Jack et al., 2018). These clinical symptoms are caused by the accumulation of pathology leading to the macrostructural disorder of the brain, of which the hippocampus atrophy is the most obvious.

The hippocampus is composed of several subfields with different histological characteristics, rather than a homogeneous structure. Hippocampal atrophy is the most significant structural biomarker of AD imaging (Ritchie et al., 2018). Differential changes in hippocampal atrophy can be relatively easily obtained from magnetic resonance imaging (MRI). The hippocampus and hippocampal subfields are found to be diversely affected in Alzheimer’s Disease (AD) and early stages of Alzheimer’s disease by neuroimaging studies (de Flores et al., 2015; Chetelat, 2018). The hippocampal atrophy of AD patients was most significantly involved subiculum and CA1 subfields (Blanken et al., 2017). Other studies have showed that there were more extensive and more evident atrophies in DG/CA3 or subiculum at the lower end of the hippocampus (de Flores et al., 2015). Studies on prodromal AD showed that the focal atrophy of CA1-2 of MCI patients is more obvious than that of normal aging patients (Jessen et al., 2010). The atrophy first appeared in the presubiculum and subiculum of the hippocampus at MCI (Carlesimo et al., 2015). However, SCD subjects are more difficult to identify from the NC because the SCD group showed that the left total hippocampal volume was small with statistically significant difference, while the right total hippocampal volume did not change significantly (van der Flier et al., 2004; Jessen et al., 2006). The atrophy of hippocampal surface is mainly in CA1, and the other regions have obvious overlap with AD (Perrotin et al., 2017; Evans et al., 2018). The atrophy of the memory-related hippocampus and hippocampal subfields is one of the earliest macroscopic features of the trajectories of Alzheimer’s disease, and has been reported in autopsies and neuroimaging studies (Braak and Braak, 1991; Frisoni et al., 2008; Mueller et al., 2011; Mak et al., 2017). To our best knowledge, there is little research on the subfield of hippocampus and relationship with memory in SCD.

We hypothesized that there may be 1) a change in the hippocampal subfields at different stages of AD in accordance to the trajectory of Alzheimer’s disease and 2) a relationship between hippocampal subfield volume and memory status (de Flores et al., 2015; Perrotin et al., 2015; Evans et al., 2018). The purpose of this study was to identify which subfields of the hippocampus differ in the trajectories of Alzheimer’s disease by magnetic resonance imaging (MRI). In addition, to determine whether individual differences on memory could be explained by structural volumes of hippocampal subfields.

## Materials and Methods

### Participants

We prepared 161 right-handed Chinese Han participants including 35 SCD patients, 43 aMCI patients and 41 AD patients, and 42 NC subjects from our databank (NCT: 02225964, 02353845, 02353884, and 03370744). The cognitive functions of all the subjects were assessed by experienced neurologists. Including the Clinical Dementia Rating Scale (CDR) (Morris, 1993), the Chinese version of the Mini-Mental State Examination (MMSE), the Beijing version of Montreal Cognitive Assessment (MoCA) (Lu et al., 2011), the auditory verbal learning test (AVLT) (Guo et al., 2007), an activities of daily living (ADL) assessment, and Hamilton depression rating scale.

The normal controls did not present cognitive decline complaints and their performance in MMSE, MoCA and AVLT were in normal range. The patients with SCD were diagnosed based on the criteria proposed by SCD-I in 2014 (Jessen et al., 2014), including (1) self-reported experience of persistent decline in memory compared to a previous state (within the last 5 years); (2) performance within the normal range on MMSE or MoCA (adjusted for age, sex, and education); (3) the Clinical Dementia Rating (CDR) score is 0. The patients were diagnosed with aMCI using the Petersen criteria (Petersen, 2004), which have been described in our previous studies (Shu et al., 2018): (a) presence of memory complaint, confirmed by an informant; (b) presence of objective memory impairment measured by MMSE, MoCA and AVLT; (c) failure reach the standard of dementia; (d) CDR score of 0.5. The inclusion criteria for SCD were based on the recent research criteria proposed by National Institute of Aging-Alzheimer’s Association (NIA-AA) criteria for clinically probable AD (Sperling et al., 2011): (a) meeting the criteria for dementia; (b) recessive and gradual onset for more than 6 months, not a sudden attack; (c) hippocampal atrophy confirmed by structural MRI; (d) CDR score is equal or greater than 1. Exclusion criteria were prior history of the activities of daily living disorder, stroke, mental disorders, cancer, drug abuse, epilepsy, brain tumors, Parkinson’s disease, encephalitis and hypoxic brain damage. All subjects underwent brain MRI examination. The detailed demographic and clinical characteristics of participants are shown in Table 1.

**Table 1:** Characteristics of the subjects.

	NC (*n* = 42)	SCD (*n* = 35)	aMCI (*n* = 43)	AD (*n* = 41)
Age (y)	64.24 ± 6.16	64.53 ± 7.29	67.47 ± 10.03	68.88 ± 7.86
Gender (M/F)	15/27	15/20	21/22	17/24
Education (y)	11.17 ± 0.75	11.83 ± 0.82	10.44 ± 0.74	9.68 ± 0.76
MMSE	27.627 ± 0.530	27.455 ± 0.582	25.016 ± 0.520^+∗^	17.782 ± 0.542^#+∗^
MoCA	25.887 ± 0.513	24.804 ± 0.563	17.780 ± 0.503^+∗^	13.514 ± 0.524^#+∗^
AVLT, immediate recall scores	9.302 ± 0.257	8.475 ± 0.282	5.858 ± 0.252^+∗^	3.588 ± 0.263^#+∗^
AVLT, delayed recall scores	10.373 ± 0.362	8.705 ± 0.397^∗^	3.226 ± 0.355^+∗^	1.121 ± 0.370^#+∗^
AVLT, recognition scores	12.039 ± 0.464	11.212 ± 0.509	6.612 ± 0.455^+∗^	3.450 ± 0.474^#+∗^

*ANOVA followed by Bonferroni post hoc analysis for age, education, MMSE, MoCA, CDR and AVLT or the Chi-square test for gender: ^∗^p < 0.05 between NC and SCD, aMCI or AD; ^+^p < 0.05 between SCD and aMCI or AD. ^#^p < 0.05 between aMCI and AD. n = number of subjects; NC, normal control group; SCD, subjectivel cognitive decline group; aMCI, amnestic mild cognitive impairment group; AD, Alzheimer’s disease; MMSE, Mini Mental Status Examination; MoCA, the Beijing version of Montreal Cognitive Assessment; AVLT, Auditory Verbal Learning Test.*

The study approved by the medical research ethics committee and the institutional review board of Xuanwu Hospital, Capital Medical University, Beijing, China. All procedures performed in studies involving human participants were in accordance with the ethical standards of the institutional and/or national research committee and with the 1964 Helsinki declaration and its later amendments or comparable ethical standards.

### Image Acquisition

The 3T magnetic resonance imaging system (MAGNETOM Trio Tim; Siemens, Erlangen, Germany) was used for image acquisition at the Department of Radiology, XuanWu Hospital, Capital Medical University. T1-weighted MRI scans were acquired at the sagittal plane by using a magnetization prepared rapid acquisition gradient echo sequence with the following parameters: TR = 1900 ms, TE = 2.2 ms, FA = 9°, inversion time (TI) = 900 ms, matrix = 256 × 256, slices = 176, thickness = 1.0 mm and Voxel size = 1 × 1 × 1 mm^3^.

### Image Processing

Structural MR Images were processed by the FreeSurfer image analysis suite, which can be downloaded free of charge from the website (version 6.0.0, http://freesurfer.net/) (Mueller et al., 2018).

**FIGURE 1:**
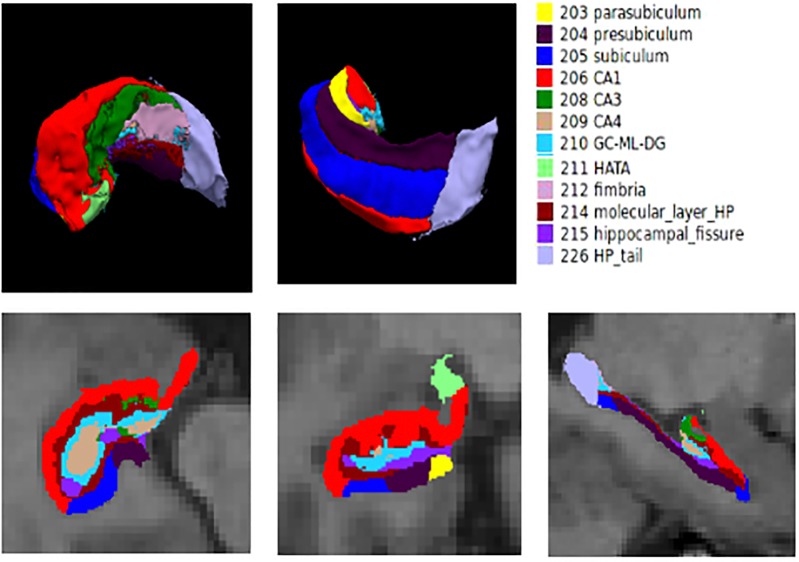
Hippocampal subfield segmentation.

First, the entire hippocampal formation was segmented using the routine volumetric FreeSurfer pipeline. Briefly, T1-weighted MR images were corrected for within-subject head motion; then, non-brain tissues were removed using a hybrid watershed/surface deformation algorithm (Segonne et al., 2004). The resulting images were further affine registered to the Talairach space. Subsequently, segmentation of the subcortical and cortical structures (including the hippocampus) was conducted using a probabilistic brain atlas (Fischl et al., 2002). The estimated total intracranial volume (eTIV) of each subject was also calculated using the standard FreeSurfer processing pipeline by exploiting the relationship between the intracranial volume and the linear transformation to the atlas template (Buckner et al., 2004). The eTIV was used to correct for individual differences in head size in the subsequent statistical analysis. Automated segmentation of hippocampal subfields was performed using a built-in module of FreeSurfer, in which a Bayesian statistical model with Markov random field priors was used to estimate the label of each subfield (Van Leemput et al., 2009). This method has been successfully applied to detect hippocampal abnormalities in specific subfields in many neuropsychiatric diseases (Kuhn et al., 2012; Haukvik et al., 2015). A bounding box containing the hippocampus that was upsampled to a 0.5 mm isotropic resolution was applied to this module. This approach relied on a tetrahedral mesh-based probabilistic atlas of the hippocampal formation, which was constructed from the manual delineation of the right hippocampus based on ultra-high-resolution T1-weighted scans (0.38 × 0.38 × 0.8 mm^3^) of 10 normal subjects. By maximizing the posterior probability of a segmentation, the left and right hippocampi were automatically segmented into twelve subfields: hippocampal tail, parasubiculum, presubiculum, subiculum, CA1, CA3, CA4, hippocampus–amygdala transition area (HATA), granule cell layer of dentate gyrus (GC-DG), molecular layer, fimbria, and hippocampal fissure. In this manuscript, the method for automated segmentation is standard. Additionally, the method for segmentation is validated to be accurate by Iglesias et al. (2015). The hippocampal subfield segmentation results are illustrated in Figure 1. The entire hippocampal volume was defined as the sum of the volume of all hippocampal subfields.

### Statistical Analysis

Statistical analysis was carried out using Statistical Package for Social Sciences software (SPSS, version 21.0). All the statistical tests were two-tailed. Categorization of demographic variables was assessed using Chi-square test. Continuous demographic variables were evaluated through ANOVA. In this study, the estimated total intracranial volume (eTIV) was used as a covariate to control head size. Statistically significant differences based on ANOVA (*P* < 0.05) were further explored using Bonferroni *post hoc* analysis. In the *post hoc* analysis, the differences between the individual experimental group and the control group were assessed. The left and right hemisphere measurements were analyzed, respectively. In addition, covariance analysis was used to analyze the volume differences in individual hippocampal subfield with age, sex, years of education and eTIV as covariates. Furthermore, we investigated relationships between hippocampal subfield volumes and memory test variables (AVLT-immediate recall, AVLT-delayed recall, AVLT-recognition) through the regression model analyses controlled for age, gender, education and eTIV.

## Results

### Demographic Data

The demographic characteristics of the normal control, the patients of SCD, the patients of aMCI and the patients of AD are shown in Table 1. Four groups of age, sex, and educational level were well-matched (*P* > 0.05 for each group comparison). Comparing SCD and NC groups, there were no significant differences in MoCA, MMSE, immediate recall part of AVLT, the recognition part of AVLT, while significant difference (*P* = 0.012) in the delayed recall part of AVLT. The patients with AD and aMCI had significant lower scores in MoCA, MMSE, and AVLT compared with the healthy control participants (*P* < 0.005).

### Comparisons of Hippocampal Subregion Volumes

**FIGURE 2:**
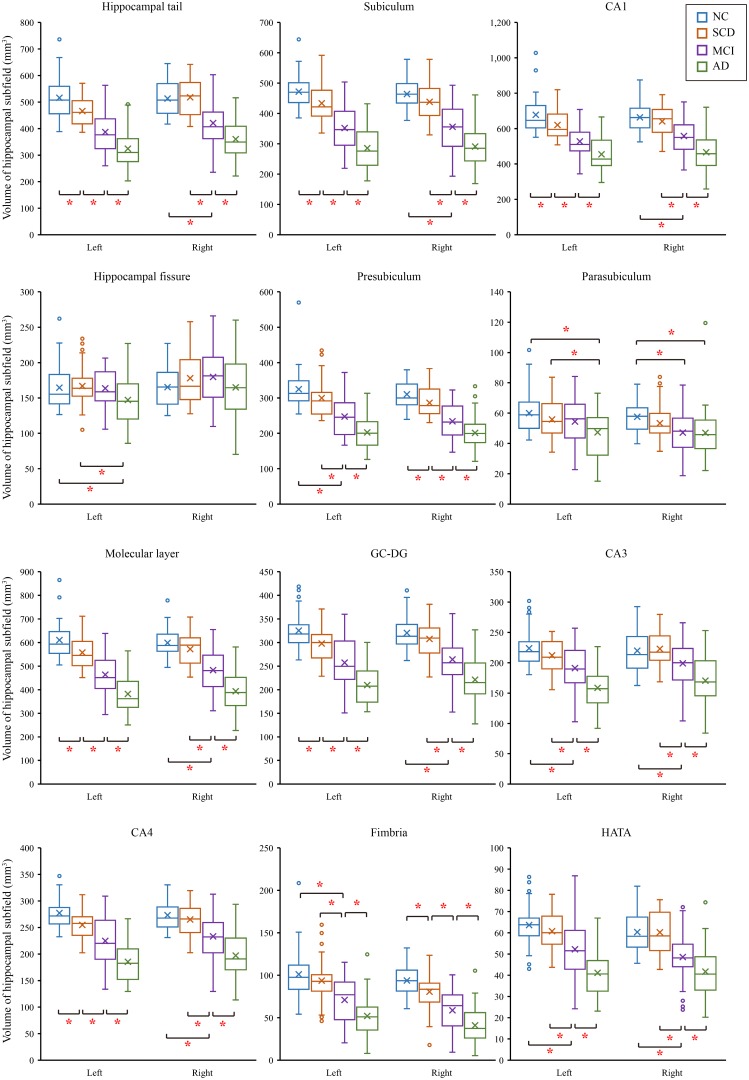
Comparison of hippocampal subregions volume in normal controls and patients with SCD, aMCI and AD. ^∗^*P* < 0.05.

**FIGURE 3:**
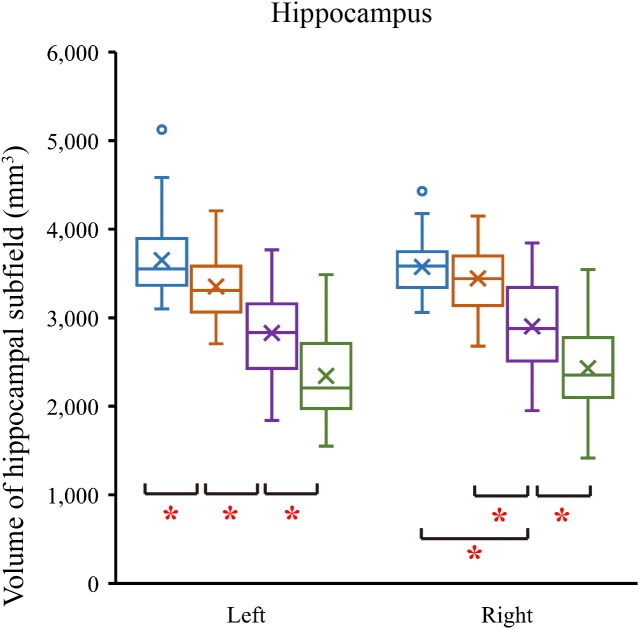
Comparison of hippocampal volume in normal controls and patients with SCD, aMCI and AD. ^∗^*P* < 0.05.

We tested differences in whole hippocampal volume and all subfields among four groups using ANCOVA with age, years of education, and eTIV as covariates. Table 2 shows the statistical results of hippocampal subfields and hippocampal volumes. The volume of the left whole hippocampus was significantly different between NC, SCD, aMCI and AD in Figure 2. However, there was no statistically significant difference in the right whole hippocampus between NC and SCD. Compared with NC, aMCI group and AD group showed significant decreases in right whole hippocampal volume in Figure 3. In addition, the significant decreases were found for SCD and NC in the volume of hippocampal tail, subiculum, presubiculum, molecular layer HP, GC-ML-DG and CA4 of left hippocampal subfields, right presubiculum and fimbria of right hippocampal subfields. Most of the hippocampal subfields showed significant volumetric difference except hippocampal fissure and left parasubiculum between aMCI and NC groups. The significant differences in the hippocampal volume were detected between the AD and NC except right hippocampal-fissure. Furthermore, in our study, CA1, subiculum, presubiculum, molecular layer and fimbria showed the trend toward significant volume reduction among four groups with the trajectories of Alzheimer’s disease.

**Table 2:** Comparison of hippocampus and hippocampal subregions volume in normal controls and patients with SCD, aMCI and AD.

	NC (*n* = 42)	SCD (*n* = 35)	aMCI (*n* = 43)	AD (*n* = 41)
	Mean ± SD	Mean ± SD	Mean ± SD	Mean ± SD
left_Whole_hippocampus	3680.289 ± 66.434	3361.059 ± 72.289	2783.291 ± 66.006	2355.177 ± 67.634
left_Hippocampal_tail	517.540 ± 10.878	466.880 ± 11.837	383.044 ± 10.808	326.011 ± 11.075
left_subiculum	474.634 ± 9.777	434.136 ± 10.639	346.972 ± 9.714	287.218 ± 9.954
left_CA1	685.699 ± 13.496	622.925 ± 14.685	517.051 ± 13.409	456.056 ± 13.740
left_hippocampal-fissure	167.325 ± 4.356	168.285 ± 4.740	161.577 ± 4.328	145.089 ± 4.434
left_presubiculum	326.734 ± 7.911	300.225 ± 8.608	243.882 ± 7.860	203.645 ± 8.054
left_parasubiculum	60.618 ± 2.092	55.852 ± 2.276	53.596 ± 2.079	47.495 ± 2.130
left_molecular_layer_HP	615.260 ± 11.668	558.698 ± 12.697	455.821 ± 11.593	384.814 ± 11.879
left_GC-ML-DG	327.924 ± 6.251	298.829 ± 6.802	253.111 ± 6.211	211.089 ± 6.364
left_CA3	226.382 ± 4.893	213.331 ± 5.325	187.852 ± 4.862	158.502 ± 4.982
left_CA4	279.731 ± 5.250	255.658 ± 5.713	220.928 ± 5.217	186.254 ± 5.345
left_fimbria	101.503 ± 3.972	93.338 ± 4.322	69.638 ± 3.946	53.117 ± 4.044
left_HATA	64.264 ± 1.732	61.186 ± 1.884	51.397 ± 1.721	40.976 ± 1.763
right_Whole_hippocampus	3602.039 ± 63.511	3446.948 ± 69.108	2852.812 ± 63.102	2453.308 ± 64.658
right_Hippocampal_tail	515.276 ± 11.044	517.343 ± 12.017	415.792 ± 10.973	364.000 ± 11.243
right_subiculum	467.121 ± 9.699	438.444 ± 10.554	349.639 ± 9.637	293.715 ± 9.874
right_CA1	670.295 ± 13.016	641.795 ± 14.163	546.620 ± 12.932	470.188 ± 13.251
right_hippocampal-fissure	168.930 ± 5.345	179.797 ± 5.816	176.393 ± 5.311	162.883 ± 5.442
right_presubiculum	311.190 ± 6.445	285.782 ± 7.013	231.129 ± 6.403	203.520 ± 6.561
right_parasubiculum	57.794 ± 2.095	53.348 ± 2.280	46.570 ± 2.082	47.303 ± 2.133
right_molecular_layer_HP	603.151 ± 11.458	572.299 ± 12.468	474.457 ± 11.384	398.113 ± 11.665
right_GC-ML-DG	323.443 ± 6.254	307.969 ± 6.805	259.101 ± 6.213	223.016 ± 6.367
right_CA3	223.040 ± 5.422	223.890 ± 5.900	195.124 ± 5.388	170.701 ± 5.520
right_CA4	276.215 ± 5.430	265.840 ± 5.908	228.857 ± 5.395	198.068 ± 5.528
right_fimbria	93.357 ± 3.230	79.545 ± 3.514	58.037 ± 3.209	43.091 ± 3.288
right_HATA	61.156 ± 1.565	60.693 ± 1.703	47.486 ± 1.555	41.594 ± 1.593

*Mean and standard deviation of subfield and total hippocampal volumes in mm^3^.*

### Relationship Between AVLT and Hippocampal Subregion Volumes

In a first step, all potential risk factors (age, education years, sex, GM volume of hippocampal subfields, TIV) were correlated with AVLT scores and only variables correlated with AVLT score at *P* < 0.2 were used in subsequent stepwise linear regressions. This was performed to avoid too many independent variables. In the regression model, variables were removed when *P* > 0.05. Table 3 presents the results of the linear regression analyses. In our study, volume of left subiculum of all the four groups was most strongly and actively correlated with performance of AVLT three measures.

**Table 3:** Linear Regression Models for Different AVLT scores.

Dependent Variable	Variables Included in the Model	Unstandardized *B*	Coefficients Standard Error	Standardized Coefficients β	*P*
	Constant	−3.814	0.948		<0.001
	Left_subiculum	0.011	0.003	0.434	<0.001
AVLT,	Sex	1.239	0.301	0.248	<0.001
immediate recall scores	Education years	0.125	0.031	0.246	<0.001
	Left_hippocampal tail	0.007	0.002	0.272	0.007
	Right_p arasubiculum	−0.028	0.013	−0.159	0.029
AVLT, delayed recall scores	Constant	−2.971	2.694		0.272
	Left_subiculum	0.011	0.005	0.240	0.021
	Education years	0.297	0.053	0.313	<0.001
	Left_hippocamal_tail	0.015	0.004	0.339	<0.001
	TIV	< 0.001	0.000	−0.153	0.006
	Right_fimbria	0.030	0.013	0.188	0.019
AVLT, recognition scores	Constant	−5.634	1.455		<0.001
	Left_subiculum	0.029	0.009	0.594	<0.001
	Education years	0.256	0.056	0.265	<0.001
	Right_fimbira	0.042	0.014	0.260	0.003
	Left_presubuiculum	−0.025	0.011	−0.370	0.025
	Right_hippocamapl_tail	0.009	0.004	0.182	0.04

*In a first step, all potential risk factors (age, education years, sex, GM volume of hippocampal subfields, TIV) were correlated with AVLT scores and only variables correlated with AVLT score at P < 0.2 were used in subsequent stepwise linear regressions. This was performed to avoid too many independent variables. In the regression model, variables were removed when P > 0.05.*

## Discussion

In this study, we investigated the volumetric difference of hippocampus and hippocampal subregions among AD, aMCI, SCD, and NC subjects. There were also trends in some hippocampal subregions with the trajectories of Alzheimer’s disease in addition to the volumetric differences between the four groups. Furthermore, we studied AVLT and typical hippocampal subfields related with memory. It also shown trends with the trajectories of Alzheimer’s disease.

In our study, we found that the differences of hippocampus and hippocampal subfields with age, years of education, and eTIV as covariates. The effect of the size of the brain in different subjects was excluded. Our study showed that the difference in volumes was in the left whole hippocampus as that of previous studies (van der Flier et al., 2004; Jessen et al., 2006). We further divided the volume of the hippocampus, and the volumetric subfields of SCD, aMCI and AD were compared with the volumetric subfields of the NC. The hippocampal subfields volume of AD had significant differences except for right hippocampal fissure. There were also volumetric differences of aMCI in hippocampal tail, subiculum, presubiculum, molecular layer HP, GC-ML-DG, CA4, CA3, fimbria, HATA and right parasubiculum. These were consistent with previous studies (Kang et al., 2018; Su et al., 2018). Previous studies had shown that the volume of the whole hippocampus and hippocampal subfields of SCD and NC were not consistent (van der Flier et al., 2004; Jessen et al., 2006; Carr et al., 2017). But our research found that the volumes of SCD were different from those of NC in left whole hippocampus hippocampal tail, subiculum, presubiculum, molecular layer HP, GC-ML-DG and CA4 of left hippocampal subregions, right presubiculum and right fimbria. Of note, we observed the trend in the CA1, subiculum, presubiculum, molecular layer and fimbria subregions, which were in line with the previous studies, but their studies rarely involved the trajectories of Alzheimer’s disease (Perrotin et al., 2015; Carr et al., 2017; Lindberg et al., 2017). The obvious atrophic structures in AD are located at CA1, subiculum and the presubiculum (Carlesimo et al., 2015). The atrophy of CA1 in MCI has also been reported, which is related to the increased risk of conversion from MCI to AD (Apostolova et al., 2006). In our study, we found that the hippocampus-related subfields had changed as early as SCD stages, however, not all of them showed trend changes. Trend-changing parts are rich in fibers and synapses, which also provide intrahippocampal connections and receive inputs from the hypothalamic lobe and thalamic nucleus. This is strongly correlated with memory impairment in AD patients (Lace et al., 2009). Our finding about the hippocampal volume reduction are consistent with neuropathological findings in the progression of AD disease (Mizutani and Kashara, 1995). In our study, the atrophies of CA1, subiculum, presubiculum, molecular layer and fimbria subregions among SCD, aMCI and AD groups suggest that they may be a potential early biomarker for detecting AD at the SCD stage. These results similarly suggest that, compared with normal control subjects, the difference in the volumes of hippocampal subfields and the trend of these changes could show the evolution of AD in the earlier stage.

The functions of the hippocampal subfield were different, which were related to memory, executive function, attention deficits and so on (Serkova et al., 2016; Evans et al., 2018). The analysis of subfield volumes has been applied to memory neuroscience suggesting that subregion such as CA1, CA3 and dentate gyrus in memory is important (Kesner, 2013; Tamnes et al., 2014; Suthana et al., 2015). In our study, the scores of delayed recalls of AVLT were more closely related to the changes of hippocampal subfields than the score of immediate memory and recognition. As we all knew delayed recalls reflect the episodic memory which was impaired first in AD. Furthermore, the scores of delayed recalls of AVLT were better correlated with left subiculum. It implied that left subiculum might tell diseases earlier as an imaging biomarker (Duara et al., 2012; Jessen et al., 2014; Tamnes et al., 2014; Suthana et al., 2015).

There are limitations in our study. Firstly, the main limitation is the lack of high risk group but asymptomatic control group besides the four groups (AD, aMCI, SCD and NC). In future design, we will collect the high risk but asymptomatic control group. Furthermore, this study was based on cross-sectional data, longitudinal follow-up studies of the same cohort are conducted to identify early imaging markers for disease transformation and prediction. Finally, we only studied hippocampal subregion volume by structural MRI. The combination of the multimodal imaging (i.e., structural, functional MR imaging and positron emission tomography technique) could be used in our future research.

## Conclusion

Our findings show that the trend changes in the hippocampus subfield and further illustrate that SCD is the preclinical stage of AD earlier than aMCI. The susceptibility of hippocampal subfield to AD pathological damage is different, so the volume of hippocampal subfield is better than the total volume of hippocampus in identifying early AD. It can better review the trajectory of AD, understand the mechanism, and identify sensitive biological indicators at different stages of AD.

## Data Availability

All datasets generated for this study are included in the manuscript and/or the supplementary files.

## Author Contributions

WZ contributed to the main body of the manuscript. YH and SL conceptualized, designed, supervised the study and approved the final version of the manuscript. CY and MH contributed to study design and data collection. XW contributed to data analysis and manuscript preparation. All the authors interpreted the data, provided important feedback, and revised the manuscript.

## Conflict of Interest Statement

The authors declare that the research was conducted in the absence of any commercial or financial relationships that could be construed as a potential conflict of interest.
